# Genetic background determines synaptic phenotypes in *Arid1b*-mutant mice

**DOI:** 10.3389/fpsyt.2023.1341348

**Published:** 2024-03-07

**Authors:** Hyosang Kim, Eunjoon Kim

**Affiliations:** ^1^Center for Synaptic Brain Dysfunctions, Institute for Basic Science (IBS), Daejeon, Republic of Korea; ^2^Department of Biological Sciences, Korea Advanced Institute of Science and Technolgoy (KAIST), Daejeon, Republic of Korea

**Keywords:** ARID1B mutation, genetic background, miniature excitatory postsynaptic currents, miniature inhibitory postsynaptic currents, synaptic phenotype

## Abstract

ARID1B, a chromatin remodeler, is strongly implicated in autism spectrum disorders (ASD). Two previous studies on *Arid1b*-mutant mice with the same exon 5 deletion in different genetic backgrounds revealed distinct synaptic phenotypes underlying the behavioral abnormalities: The first paper reported decreased inhibitory synaptic transmission in layer 5 pyramidal neurons in the medial prefrontal cortex (mPFC) region of the heterozygous *Arid1b*-mutant (*Arid1b*^+/−^) brain without changes in excitatory synaptic transmission. In the second paper, in contrast, we did not observe any inhibitory synaptic change in layer 5 mPFC pyramidal neurons, but instead saw decreased excitatory synaptic transmission in layer 2/3 mPFC pyramidal neurons without any inhibitory synaptic change. In the present report, we show that when we changed the genetic background of *Arid1b*^+/−^ mice from C57BL/6 N to C57BL/6 J, to mimic the mutant mice of the first paper, we observed both the decreased inhibitory synaptic transmission in layer 5 mPFC pyramidal neurons reported in the first paper, and the decreased excitatory synaptic transmission in mPFC layer 2/3 pyramidal neurons reported in the second paper. These results suggest that genetic background can be a key determinant of the inhibitory synaptic phenotype in *Arid1b*-mutant mice while having minimal effects on the excitatory synaptic phenotype.

## Introduction

ARID1B (AT-rich interaction domain 1B; also known as BAF250B) is a subunit of the SWI/SNF chromatin remodeling complex implicated in various neurodevelopmental, psychiatric, and cognitive disorders, including autism spectrum disorders (ASD), intellectual disability, and Coffin-Siris syndrome (1–6).

Previous studies on *Arid1b*-mutant mice reported various ARID1B deficiency-related phenotypes and underlying mechanisms (7–10), including suppressed insulin-like growth factor signaling (8), impaired inhibitory synaptic transmission (9), and impaired excitatory synaptic transmission (10). The impaired inhibitory synaptic transmission was observed among layer 5 pyramidal neurons in the medial prefrontal cortex (mPFC) of heterozygous *Arid1b*-mutant (*Arid1b*^+/−^) mice that lacked exon 5 of the *Arid1b* gene (9). In a similar and more recent study on *Arid1b*^+/−^ mice also lacking exon 5, our group did not find inhibitory synaptic changes in layer 5 pyramidal mPFC neurons, but rather observed impaired excitatory synaptic transmission in layer 2/3 mPFC pyramidal neurons (10). This discrepancy in the synaptic phenotypes of two *Arid1b*^+/−^ mouse lines with the same exon 5 deletion may reflect a difference in the genetic background of the mutant mouse lines: The first study used C67BL6/J mice, while we used C67BL/N mice. These genetic backgrounds have been reported to exhibit multiple phenotypic differences, including differences in synaptic transmission and metabolism (11–14).

In the present study, we attempted to change the genetic background of our *Arid1b*^+/−^ mice from C57BL/6 N to C57BL/6 J by backcrossing with wild-type C57BL/6 J mice for more than seven generations. We found that our new *Arid1b*^+/−^ mice with the C57BL/6 J background show impaired inhibitory synaptic transmission in layer 5 mPFC pyramidal neurons, similar to the previous results published by the other group (9). However, our new *Arid1b*^+/−^ mice with the C57BL/6 J background continued to show impaired excitatory synaptic transmission in layer 2/3 mPFC pyramidal neurons, similar to our previous results (10). These findings suggest that genetic background is a key determinant of the inhibitory synaptic phenotype in *Arid1b*^+/−^ mice carrying exon 5 deletion, whereas the excitatory synaptic phenotypes are minimally affected by the genetic background.

## Results

### Synaptic transmission differs between two exon 5-deleted *Arid1b*^+/−^ mouse lines

**Figure 1:**
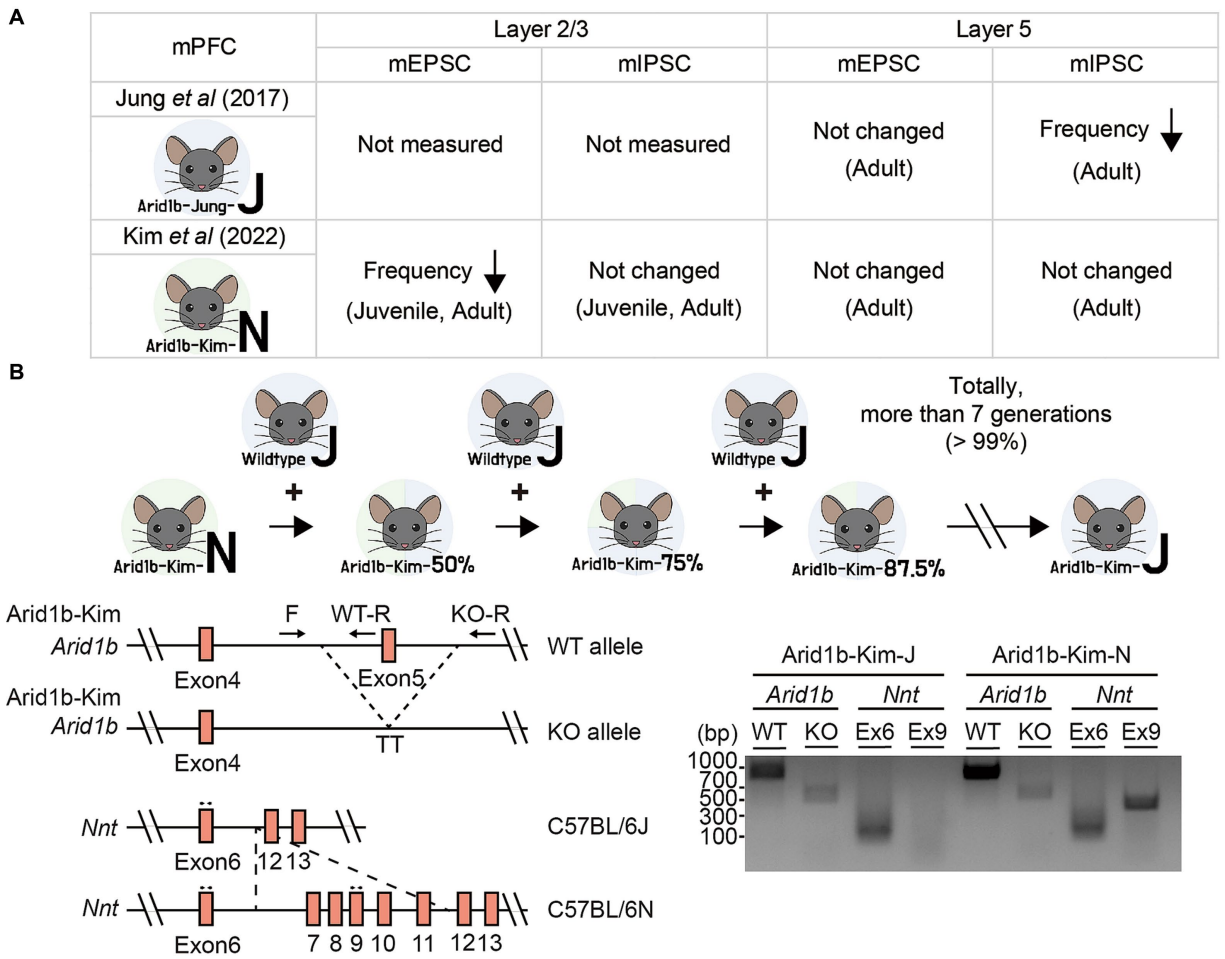
Genetic backgrounds of *Arid1b*^+/−^ mice and their changes. **(A)** Summary of the electrophysiological properties of layer 2/3 or layer 5 pyramidal neurons in mPFC regions representing two *Arid1b*^+/−^ mouse lines of different genetic backgrounds (C57BL/6 J and C57BL6/N) having the same exon 5 deletion, as reported in two different studies (termed Arid1b-Jung-J and Arid1b-Kim-N mice, respectively). mEPSC, miniature excitatory postsynaptic currents; mIPSC, miniature inhibitory postsynaptic currents. **(B)** (Upper) A schematic diagram showing strategy of backcrossing Arid1b-Kim-N mice with wild-type (WT) C57BL/6 J mice for more than seven generations to generate Arid1b-Kim-J mice. (Lower left) Schematic diagrams of the WT and mutant Arid1b alleles and the Nnt alleles of the C57BL/6 N and C57BL/6 J backgrounds, along with the primers used to genotype them. (Lower right) PCR genotyping results indicating that the genetic background was successfully switched from C57BL/6 N to C57BL/6 J, as tracked by the lack of *Nnt* exon 9 in the C57BL/6 J background.

Two previous studies on *Arid1b*^+/−^ mice (exon 5 deletion) reported distinct changes among excitatory and inhibitory synaptic transmissions in layer 2/3 or 5 pyramidal neurons in the mPFC (Figure 1A). Specifically, a previous study on *Arid1b*^+/−^ mice in the genetic background of C57BL/6 J (EUCOMM; MGI:4435087) reported that the frequency but not amplitude of miniature inhibitory postsynaptic currents (mIPSCs) was decreased in layer 5 pyramidal neurons in the mPFC while excitatory miniature excitatory postsynaptic currents (mEPSCs) were not altered (9). This functional change was observed in adult brains and further supported by a decrease in the excitatory synapse number, as revealed by electron microscopy (EM) (9).

In contrast, our more recent study on *Arid1b*^+/−^ mice with the same exon 5 deletion in the genetic background of C57BL/6 N (NorCOMM2; MGI:6156423) revealed normal mIPSCs and mEPSCs in layer 5 mPFC pyramidal neurons but a decrease in the frequency (not amplitude) of mEPSCs in layer 2/3 pyramidal neurons without changes in mIPSCs (10). These excitatory synaptic changes were supported by EM results and observed at both juvenile and adult stages (10).

We hypothesized that the difference in the genetic backgrounds of the *Arid1b*^+/−^ mouse lines (hereafter termed “Aridb1-Jung-J” and “Arid1b-Kim-N” mice) might contribute to the discrepancy in the observed synaptic phenotypes. To test this hypothesis, we attempted to change the genetic background of our *Arid1b*^+/−^ mice from C57BL/6 N to C57BL/6 J (i.e., Arid1b-Kim-N mice to Arid1b-Kim-J mice) by backcrossing Arid1b-Kim-N mice with wild-type (WT) C57BL/6 J mice for more than seven generations (Figure 1B). This change in the genetic background was confirmed by PCR genotyping for exon 9 of the *Nnt* (nicotinamide nucleotide transhydrogenase) gene (14), which was present in Arid1b-Kim-N mice but absent from Arid1b-Kim-J mice.

### Synaptic transmission in Arid1b-Kim-J mice

**Figure 2:**
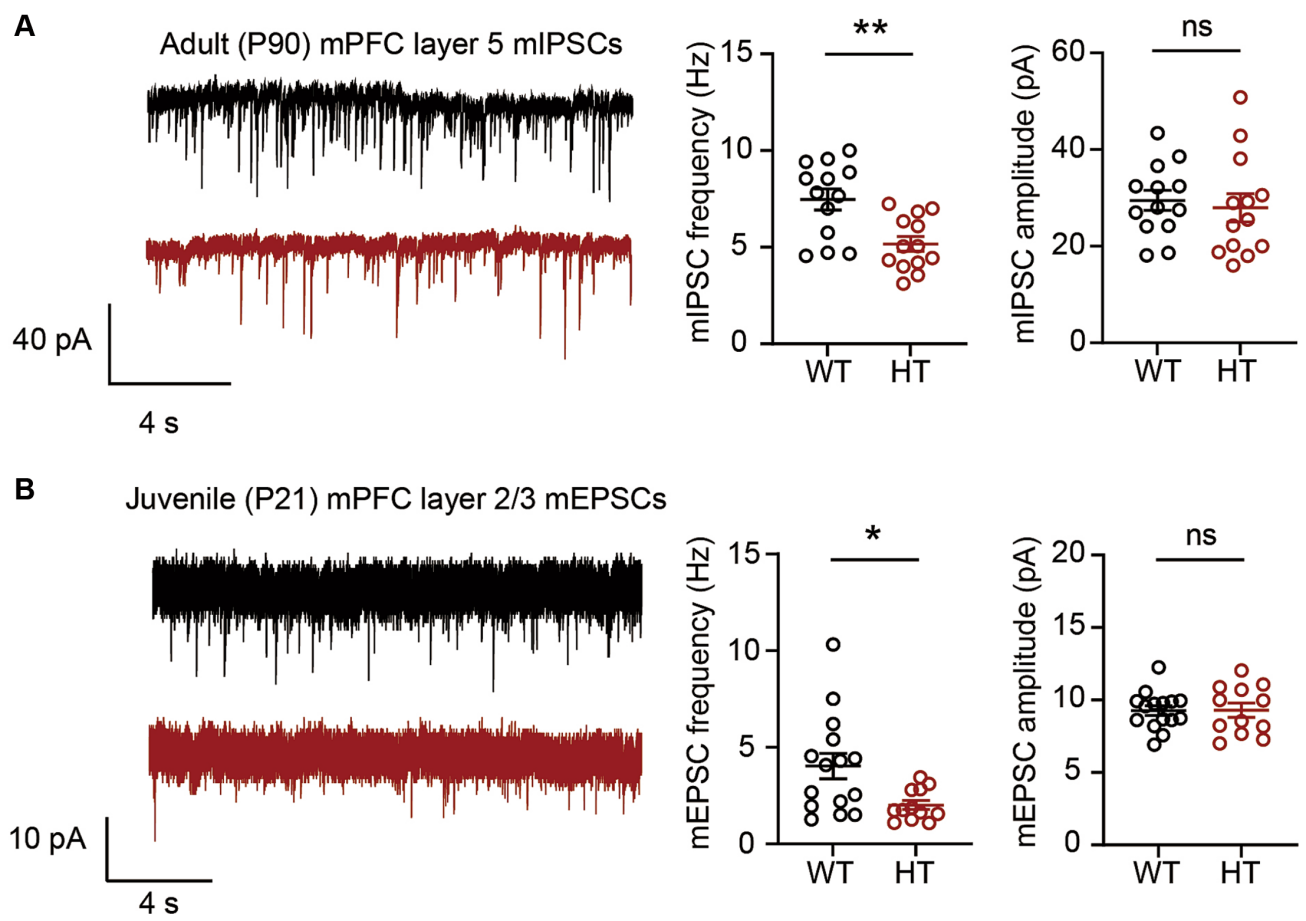
Excitatory and inhibitory synaptic phenotypes in Arid1b-Kim-J mice. **(A)** Decreased frequency but normal amplitude of miniature inhibitory postsynaptic currents (mIPSCs) among layer 5 pyramidal neurons in the prelimbic region of the medial prefrontal cortex (mPFC) from adult (postnatal day [P] 90) Arid1b-Kim-J mice (*n* = 13 neurons from 3 mice for WT, 13 neurons from 3 mice for HT; Student’s *t*-test, **p < 0.01, ns, not significant). **(B)** Decreased frequency but normal amplitude of miniature excitatory postsynaptic currents (mEPSCs) among layer 2/3 pyramidal neurons in the prelimbic region of the mPFC from juvenile (P19–21) Arid1b-Kim-J mice (*n* = 15 neurons from 3 mice for WT, 12 neurons from 3 mice for HT; Student’s *t*-test, *p < 0.05, ns, not significant).

We then measured mIPSCs in Arid1b-Kim-J mice to see if the shift of genetic background from C57BL/6 N to C57BL/6 J had any effect on inhibitory synaptic transmission in layer 5 mPFC pyramidal neurons at postnatal day 90 (P90), when mIPSCs were measured in the first study (9). Similar to the previous results (9), we observed a decrease in the frequency but not amplitude of mIPSCs in layer 5 mPFC neurons of Arid1b-Kim-J mice (Figure 2A). This suggests that *Arid1b* haploinsufficiency in mice leads to inhibitory synaptic deficits in the C57BL/6 J background but not the C57BL/6 N background.

We next tested if the mEPSCs found to be suppressed in the Arid1b-Kim-N mice (10) were affected by the change of genetic background from C57BL6/N to C57BL6/J. Measurements of mEPSCs in layer 2/3 mPFC pyramidal neurons in Arid1b-Kim-J mice at P21 revealed a decrease in the mEPSC frequency but not the amplitude (Figure 2B), which was similar to the results we previously obtained in P21 Arid1b-Kim-N mice (10). This suggests that excitatory synaptic transmission deficits are consistently observed in *Arid1b*^+/−^ mouse lines of the two different genetic backgrounds (Arid1b-Kim-N and Arid1b-Kim-J mice).

## Discussion

In this study, we demonstrate that the genetic background has strong effects on inhibitory synaptic transmission but not excitatory synaptic transmission in *Arid1b*^+/−^ mice. This explains the distinct synaptic phenotypes observed in two previously reported *Arid1b*^+/−^ mice having the same exon 5 deletion in different genetic backgrounds: Decreased mIPSC frequency was reported in Arid1b-Jung-J mice but decreased mEPSC frequency was found in Arid1b-Kim-N mice. The results from the present study suggest that genetic background of *Arid1b*^+/−^ mice can modulate an excitation/inhibition imbalance, a mechanism implicated in ASD (15–19), through the regulation of inhibitory synaptic transmission.

It remains unclear how altering the genetic background of *Arid1b*^+/−^ mice from C57BL/6 N to C57BL/6 J can decrease inhibitory synaptic transmission. We do know that mice of C57BL/6 N and C57BL/6 J backgrounds differ in their genetic and phenotypic profiles (12–14). Genetic differences between C57BL/6 N and C57BL/6 J mice include 34 single-nucleotide polymorphisms, 2 indels, and 15 structural variants. Phenotypic differences were observed in various domains, including dysmorphology, ophthalmology, cardiovascular, metabolism, neurological, behavioral, sensory, clinical chemistry, hematology, and immune/allergy.

Previous studies on Arid1b-Jung-J mice showed that Arid1b haploinsufficiency dramatically decreases the number of parvalbumin-positive GABAergic interneurons through various mechanisms, including altered Wnt/β-catenin signaling and impaired modulation of histone acetyltransferase activity in the promotor region of the parvalbumin/*Pvalb* gene (9, 20). We speculate that the genetic background switch from Arid1b-Kim-N to Arid1b-Kim-J mice might have altered these mechanisms involved in parvalbumin-positive GABA neuronal development. With regard to a candidate mechanism, wild-type mice of C57BL/6 N background carry a mutation (S968F) in the CYFIP2 (cytoplasmic FMR1-interacting protein 2) protein (11). CYFIP2 is a component of the actin regulatory WAVE complex, which binds the fragile X messenger ribonucleoprotein (FMRP) (21–23) and has been implicated in developmental delay, epilepsy, West syndrome (a developmental and epileptic encephalopathy), and Alzheimer’s disease (23–25). The CYFIP2-S968F mutation was originally reported to destabilize the protein (11), but later shown to also induce excessive Rac1-mediated WAVE complex activation (26). In addition, *in vitro* and *in vivo* studies have shown that CYFIP2 regulates various neuronal and synaptic functions, including neuronal excitability, excitatory and inhibitory synaptic structure/function, presynaptic/mitochondrial function, neuronal excitability, and local translation (11, 23, 27–33). In the context of the present work, it is notable that CYFIP2 is enriched at inhibitory synapses, and its neuronal overexpression suppresses inhibitory synaptic structure (30). Thus, we speculate that the loss of the CYFIP2-S968F mutation in Arid1b-Kim-J mice during the genetic background switch might have contributed to inducing inhibitory synaptic deficits. Future studies will be needed to explore this possibility in detail.

Secondly, *Arid1b* haploinsufficiency in mice has been shown to cause growth impairment and deficiency of insulin-like growth factor (IGF) (8); this key regulator of brain/neuronal/synaptic development and metabolism has been implicated in various brain disorders, including ASD and neurodegenerative disorders (34–38). IGF-1, in particular, has been shown to induce long-lasting changes of inhibitory synaptic transmission in the neocortex, cerebellum, and olfactory bulb (39–42). WT C57BL/6 J mice reportedly show distinct metabolic states (body weight, tissue weight, and plasma insulin levels) compared to those of WT C57BL6/N mice (43). This is thought to involve differential gene expression and spontaneous genetic mutations. For example, deletion of exons 7–11 from the *Nnt* gene in C57BL/6 J mice is known to induce metabolic changes, such as glucose intolerance and body weight changes (44–46). We therefore suggest that C57BL/6 J-specific metabolic changes might have interfered with the *Arid1b* haploinsufficiency-induced changes of IGF signaling to induce the inhibitory synaptic deficits observed in Arid1b-Kim-J mice.

In summary, we herein show that the genetic background is a key determinant of inhibitory synaptic phenotype in cortical neurons from Arid1b-mutant mice of C57BL/6 N and C57BL/6 J genetic backgrounds, whereas the excitatory synaptic phenotype is minimally affected by these genetic backgrounds.

## Methods

### Animals

Experimental procedures using mice (*Mus musculus*) were approved by the Committee on Animal Research at KAIST (KA2020-51 and KA2023-092-v1) and performed in compliance with all relevant ethical regulations. Mice were maintained according to the Requirements of Animal Research at KAIST, fed *ad libitum*, and housed under a 13:00–01:00 dark/light cycle.

As part of the NorCOMM2 project (TCPC317) funded by Genome Canada and the Ontario Genomics Institute (OGI-051) at the Toronto Centre for Phenogenomics, *Arid1b*^+/−^ mice were generated by targeting exon 5 of *Arid1b* using CRISPR/Cas9 (C57BL/6 N-Arid1b^em1Tcp^). This involved the use of Cas9 nickase (D10A) and single-guide RNAs with spacer sequences CTGCTTAGCAAGTTACCACT and GCCTGATACAGCACTTACAT for targeting the 5′ side of exon OTTMUSE00000314956 (exon 5), and sequences ACACTAAAGGGGTTGCTTTC and CTTGTAATCCCCCTGTAGTA for targeting the 3′ side. This resulted in deletion of Chr17 from 5242523 to 5243410 with insertion of “TT.” For the present work, these mice were obtained from the Canadian Mouse Mutant Repository.

The originally published *Arid1b*-mutant mice were maintained in the C57BL/6 N background (10). To change the genetic background from C57BL/6 N to C57BL/6 J, male *Arid1b*^+/−^ mice of C57BL/6 N background were backcrossed with female wild-type C57BL/6 J mice for more than seven generations. All mice used in the present study were generated by crossing backcrossed male *Arid1b*^+/−^ mice of the C57BL/6 J genetic background with female wild-type C57BL/6 J mice. Pups were weaned at postnatal day (P) 21. After weaning, three to six mice of the same genetic background were co-housed in a single cage. The following primers were used for PCR genotyping: Arid1b HT/heterozygotes, F/forward CATTACAGTGTCCTCTCCCATCTTG and WT-R/reverse GAAAGAGAAAGCGGGTGTTCATAC plus knockout (KO)-R CGGTGTGTGACTGTGATCATAGATG; for Nnt, primer ex6 F GGGTTTCGATTGCTGTCATT and ex6 R AGTCAGCAGCACTCCTCCAT; and ex9 F CCAGCATGCACTCTCTTCTG and ex9 R TGGTCTCCAAGTGCACAGAG (47).

### Electrophysiology

For electrophysiological recordings, mice were anesthetized using isoflurane (Terrell). To prepare coronal mPFC sections, extracted brains were sectioned (300 μm) using a vibratome (Leica VT1200) in ice-cold sucrose based cerebrospinal fluid buffer containing (in mM) 212 sucrose, 25 NaHCO_3_, 10 D-glucose, 5 KCl, 2 Na-pyruvate, 1.25 L-ascorbic acid, 1.25 NaH_2_PO_4_, 3.5 MgSO_4_, and 0.5 CaCl_2_ bubbled with 95% O_2_ and 5% CO_2_ gas. Sectioned brain slices were recovered at 32°C in a chamber loaded with artificial cerebrospinal fluid (ACSF) containing (in mM) 125 NaCl, 25 NaHCO_3_, 2.5 KCl, 1.25 NaH_2_PO_4_, 1.3 MgCl_2_, and 2.5 CaCl_2_ and bubbled with 95% O_2_ and 5% CO_2_ gas. After 30 min, the chamber was moved to room temperature and a second 30-min recovery was performed with bubbling of 95% O_2_ and 5% CO_2_ gas. For recordings, slices were moved to a recording chamber perfused with circulating ACSF at 28°C. Borosilicate glass pipettes (Harvard Apparatus) were pulled using an electrode puller (Narishige) and used as recording pipettes. For whole-cell recordings, recording pipettes (2.5–3.5 MΩ) were filled with the following internal solutions: (1) for EPSC experiments, in mM, 117 CsMeSO_4_, 10 TEA-Cl, 8 NaCl, 10 HEPES, 5 QX-314-Cl, 4 Mg-ATP, 0.3 Na-GTP, 10 EGTA with pH 7.3, and 285–300 mOsm; and (2) for IPSC experiments, in mM, 115 CsCl_2_, 10 TEA-Cl, 8 NaCl, 10 HEPES, 5 QX-314-Cl, 4 Mg-ATP, 0.3 Na-GTP, 10 EGTA with pH 7.3, and 285–300 mOsm. Miniature synaptic currents were measured while holding the voltage at −70 mV. The responses were filtered at 2 kHz and digitized at 10 kHz (MultiClamp 700B and DigiData 1550, both from Molecular Devices). Data files were acquired using pClamp (10.1, Molecular Devices) and analyzed using Clampfit 10 (Molecular Devices). Tetrodotoxin (10 μM, Abcam) was added to the ACSF to inhibit action potential firing during miniature current recordings. For mEPSC measurements, picrotoxin (100 μM, Abcam) was added to the ACSF to block inhibitory synaptic currents. For mIPSC measurements, NBQX (100 μM, Tocris) and D-AP5 (100 μM, Tocris) were added to the ACSF to block AMPA and NMDA receptor-mediated currents, respectively.

### Statistical analysis

Data are presented as means with standard error of mean (SEM). Statistical analyses were performed using GraphPad Prism software. Details on mice and statistical details are summarized in Supplementary Table S1.

## Data availability statement

The original contributions presented in the study are included in the article/Supplementary material, further inquiries can be directed to the corresponding author.

## Ethics statement

The animal study was approved by Committee on Animal Research at KAIST. The study was conducted in accordance with the local legislation and institutional requirements.

## Author contributions

HK: Data curation, Investigation, Writing – original draft. EK: Conceptualization, Data curation, Funding acquisition, Project administration, Resources, Supervision, Validation, Writing – original draft, Writing – review & editing.
